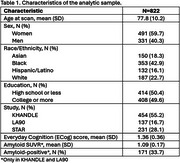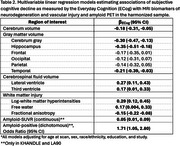# Associations of subjective cognitive decline with MRI biomarkers of neurodegeneration and vascular injury and amyloid PET in an ethnoracially diverse cohort

**DOI:** 10.1002/alz70857_105950

**Published:** 2025-12-25

**Authors:** Nancy X Chen, Sarah Tomaszewski Farias, Alexander Ivan B. Posis, Batool M. Rizvi, Paola Gilsanz, María M. M. Corrada, M. Maria Glymour, Elizabeth Rose Mayeda, Rachel A. Whitmer

**Affiliations:** ^1^ University of California, Davis, Davis, CA, USA; ^2^ University of California, Davis School of Medicine, Sacramento, CA, USA; ^3^ Kaiser Permanente Northern California Division of Research, Pleasanton, CA, USA; ^4^ University of California, Irvine, Irvine, CA, USA; ^5^ Boston University School of Public Health, Boston, MA, USA; ^6^ UCLA Fielding School of Public Health, University of California, Los Angeles, CA, USA

## Abstract

**Background:**

Subjective cognitive decline can be an early indicator of underlying brain pathology. We investigated associations of subjective cognitive decline with imaging biomarkers of neurodegeneration, cerebrovascular injury, and amyloid burden.

**Method:**

Harmonized sample of ethno‐racially diverse study participants from Kaiser Healthy Aging and Diverse Life Experiences (KHANDLE), *LifeAfter90* (LA90), and the Study of Healthy Aging in African Americans (STAR) aged 50‐101. Subjective cognitive decline was measured via the 12‐item Everyday Cognition (ECog) scale (range=1‐4), where higher scores suggest more subjective cognitive decline. Regional brain volumes and white matter integrity were measured using 3T MRI. Amyloid was measured using florbetapir PET among a random subset of KHANDLE and LA90. Measures of cerebrum and gray matter volume (cerebrum gray, hippocampus, frontal, occipital, parietal, temporal), cerebrospinal fluid volume (lateral, third), and log‐white matter hyperintensities were normalized with intracranial volume. We also examined free water fraction and fractional anisotropy. All MRI measures were z‐standardized. Amyloid burden was quantified using standard uptake value ratios (SUVR) and amyloid positivity was defined as SUVR ≥1.1. Linear regression and logistic models estimated associations of subjective cognitive decline with global and regional brain volumes and white matter injury, SUVR, and amyloid‐positivity. All models adjusted for sex, race/ethnicity, age, education, and cohort.

**Result:**

Participants’ (*N* = 822) mean±SD age was 77.8±10.2, 60% were women, 50% had at most a high school education and 18% identified as Asian, 43% as Black, 16% as Hispanic/Latino (Table 1). Overall, the mean ECog was 1.36±0.36, mean amyloid SUVR was 1.09±0.17, and 33.7% were amyloid‐positive). Higher (worse) ECog was associated with smaller volumes in cerebrum (β=‐0.18,95%CI=‐0.31,‐0.05), cerebrum gray (β=‐0.30,95%CI=‐0.47,‐0.13), hippocampus (β=‐0.35,95%CI=‐0.51,‐0.18), and temporal cortex (β=‐0.21,95%CI=‐0.39,‐0.03); larger volumes in lateral (β=0.27,95%=CI 0.11,0.43) and third ventricles (β=0.17,95%CI 0.01,0.33); greater log‐white matter hyperintensities (β=0.29,95%CI=0.12,0.45) and free water (β=0.17,95%CI=0.004,0.33); and lower fractional anisotropy (β=‐0.15,95%CI ‐0.22,‐0.08) (Table 2). Greater ECog was also associated with higher amyloid‐SUVR (β=0.05,95%CI 0.01,0.09) and higher odds of amyloid‐positivity (OR=1.71,95%CI 1.05,2.80) (Table 2).

**Conclusion:**

In this diverse cohort of older adults, greater subjective cognitive decline was associated with worse late‐life brain health. These findings highlight the importance of addressing subjective cognitive decline as potential indicators of neurodegeneration, cerebrovascular injury, and amyloid burden.